# Using mixture cure models to address algorithmic bias in diagnostic timing: autism as a test case

**DOI:** 10.1093/jamiaopen/ooaf148

**Published:** 2025-11-04

**Authors:** Peng Wu, Naomi O Davis, Matthew M Engelhard, Geraldine Dawson, Benjamin A Goldstein

**Affiliations:** Department of Biostatistics and Bioinformatics, Duke University School of Medicine, Durham, NC, 27705, United States; Department of Psychiatry, Duke University School of Medicine, Durham, NC, 27705, United States; Department of Biostatistics and Bioinformatics, Duke University School of Medicine, Durham, NC, 27705, United States; Department of Psychiatry, Duke University School of Medicine, Durham, NC, 27705, United States; Department of Pediatrics, Duke University School of Medicine, Durham, NC, 27705, United States; Department of Biostatistics and Bioinformatics, Duke University School of Medicine, Durham, NC, 27705, United States; Department of Pediatrics, Duke University School of Medicine, Durham, NC, 27705, United States

**Keywords:** mixture cure models, algorithmic bias, autism spectrum disorder, electronic health records and claims data, clinical prediction models

## Abstract

**Objectives:**

To address algorithmic bias in clinical prediction models related to the timing of diagnosis, we evaluated the efficacy of mixture cure models that integrate time-to-event and binary classification frameworks to predict diagnoses.

**Materials and Methods:**

We conducted a simulation and analyzed real-world North Carolina Medicaid data for children born in 2014, followed until 2023. The study evaluated traditional time-to-event and classification models against mixture cure models under scenarios with varied diagnostic timing and censoring.

**Results:**

Simulation results demonstrated that traditional models exhibit increased bias as diagnosis timing differences widened, whereas mixture cure models yielded unbiased estimates across varying censoring times. In real-world analyses, significant racial and ethnic variations in autism diagnosis rates were observed, with non-Hispanic White children having higher diagnosis rates compared to other groups. The mixture cure model effectively adjusted for these disparities, providing fairer and more accurate diagnostic predictions across varying levels of censoring.

**Discussion:**

Mixture cure models effectively address algorithmic bias by providing unbiased estimates regardless of variations in diagnostic timing and censoring, making them particularly suitable for conditions like autism where not all individuals will receive a diagnosis. This approach shifts focus from when an event will occur to whether it will occur, aligning more closely with clinical needs in early detection of pediatric developmental conditions.

**Conclusion:**

Mixture cure models offer a promising tool to enhance accuracy and fairness in predictive modeling, especially when the outcome of interest is not uniformly observed across groups.

## Introduction

Real-world data such as electronic health records (EHR) and administrative claims data directly reflect the way health care is delivered and received. This can be an asset when developing clinical prediction models (CPMs), but it can also be a liability. As a positive, it means the developed model should be more readily implementable as the data are reflective of normal data flows. However, as has been widely noted, these “normal” data flows often reflect societal biases, which learned CPMs could perpetuate, resulting in algorithmic bias.^1–3^ In particular, when outcomes are differentially observed across demographic groups, algorithmic bias can ensue.^4^

In many CPM scenarios, it is natural to think about the outcome as a time-to-event (TTE) outcome (eg, predicting time to death), and thus, using corresponding models like Cox Proportional Hazards (PH) and accelerated failure time (AFT) models is common.^5–7^ These methods have both substantive and analytic benefits. Substantively, it is often meaningful to know the likelihood that an event will happen earlier (ie, knowing someone will die in the next month vs next year). Analytically, TTE models allow one to easily handle common scenarios like censoring and loss to follow-up. To model a dataset in a classification framework in the presence of censoring, one needs to either ignore unequal follow-up and make the outcome “ever-never” or apply an arbitrary cut-point in time by which the outcome occurs. Neither is a desirable solution, highlighting the appeal of TTE models.

However, in some scenarios, such as the diagnosis of an underlying condition, the substantive question is not *when* the event occurs but *whether* it occurs at all. Even though the TTE framework is analytically appealing, as we will show, these models can induce algorithmic bias because it explicitly models event timing. In this study, we use the term “algorithmic bias” to refer specifically to systematic differences in predicted diagnosis probabilities that arise from group differences in diagnostic timing rather than true differences in underlying diagnosis rates. Take for example our use-case, developing a CPM to identify children at higher likelihood of receiving a diagnosis of autism spectrum disorder (“autism”). Given well-documented challenges and disparities in access to diagnostic services, the timing of diagnosis is strongly influenced by these differences across racial and ethnic groups, which creates disparities in when children receive an autism diagnosis.^8–11^ TTE models can therefore yield biased probability estimates when diagnostic timing differs between groups.

On the other hand, an alternative is to treat the outcome as purely binary (ever vs never diagnosed), which avoids the timing issue and directly aligns with the clinical questions of interest. In autism diagnosis, the goal is to predict whether an individual will ultimately be diagnosed with autism or not. Therefore, it is natural to view the outcome as binary rather than as a TTE outcome. However, binary classification models are limited because they fail to account for censoring and effectively treat incomplete follow-up as if the event will never occur. This simplification often leads to underestimation of event probabilities, particularly when many individuals have not yet been diagnosed. Thus, while classification models avoid the pitfalls of timing differences, they introduce their own source of bias by ignoring censoring. This leaves the analyst in a conundrum.

In this paper, we highlight the utility of the mixture cure model, which combines the benefits of both the classification and TTE frameworks and is advantageous when we wish to make binary predictions but must learn from TTE outcomes. After describing the mixture cure model, we first conduct a simulation study to show how it generates a less biased estimate of probability of the outcome when differences in diagnostic timing exist. We next apply this model to a real-world dataset to predict whether a child will receive an autism diagnosis. We assess differences by race and ethnicity groups, aiming to validate the model’s efficacy in delivering less biased results in real-world settings.

## Methods

### Mixture cure model

One of the underlying assumptions of TTE models is that, given enough follow-up time and no competing events, all individuals in the dataset will experience the event. The only reason not everyone is observed to experience the event is that there is censoring. This framework is applicable when modeling time to an event such as death or cancer. Given enough follow-up time, and no competing events, these will be experienced by an individual. However, there are some outcomes that not all people will experience. Autism, and other neurodevelopmental conditions, will not present in all children, no matter how long they are followed. This violates one of the primary premises of TTE models.

The mixture cure model is a parametric mixture model that was designed to resolve this problem, but it is not widely used. Motivated from cancer research to account for scenarios where patients have been cured and cannot relapse,^12^^,^^13^ it combines a TTE (“survivor”) likelihood with a classification (“cure”) one.

Let T denote the time until the event occurs. The function 1-π(z) represents the probability that a patient is cured, contingent on variables z. S(t|x) represents the survival probability at time t for those patients who are not cured, with this probability dependent on another, potentially overlapping, set of covariates x. In this framework, the mixture cure model describes the overall survival process as:


$$S_{\mathrm{pop}}(t|x,\;z)=1-\pi (z)+\pi (z)S(t|x)$$

where $S_{\mathrm{pop}}(t|x,\;z)$ is the survival function of the entire population at time t, π(z) is the proportion of the population that is susceptible to the event, called the “incidence,” and S(t|x) is the survival function for the non-cured (susceptible) subpopulation, called the “latency.” Typically, a Cox proportional hazards (Cox PH) or AFT model is employed to model the latency.

In this framework, some fraction of the population is expected to not experience the event (the cured) while the remainder are at risk (the survivors). For example, in the context of autism diagnosis, individuals who will never receive a diagnosis can be considered part of the “cured” group, whereas those who may eventually receive a diagnosis belong to the “susceptible” group. The parameter vector can be unique or shared across each component. In epidemiological studies, the focus is typically on the factors that affect survivorship, while accounting for the fact that some people have no probability of experiencing the event. In our prediction setting it is the opposite. We care about estimating the probability that someone, based on a set of characteristics, will experience the event, while ignoring the exact timing of the event.

### Simulation study

#### Design

To highlight the potential for algorithmic bias in using traditional TTE and classification models, as well as the potential of the mixture cure model to mitigate algorithmic bias, we conducted an illustrative simulation analysis motivated by our real data analysis. Each simulation was replicated 100 times, with datasets of size 10 000. An exposure variable of interest with 2 levels (eg, sex) was generated, assigning 50% to each group.

Two event-rate scenarios were considered: a “high” event rate of 25% and a “low” event rate of 2.5%. The high rate was chosen as a common setting for methodological exploration, while the low rate was selected to more closely match the low prevalence of autism diagnosis observed in our real-world dataset. In the high event-rate scenario, 75% of individuals were expected to never experience the event and were considered cured. In the low event-rate scenario, 97.5% of individuals were expected to be cured. In both scenarios, final event rates were equal across groups.

Within each event-rate scenario, we further varied diagnostic timing between groups. Event times were generated from a log-normal distribution. For group 1, the baseline median time (λ) was fixed at 4, approximating the median age of autism diagnosis (4 years old) reported in prior studies.^14^ For group 2, the median time ($\lambda_{2}$) was varied from 4 up to 10. Specifically, $\lambda_{2}\;$= 4 represented no difference in timing between groups, while progressively larger values reflected increasingly delayed diagnoses in group 2.

Finally, censoring times were generated from a uniform distribution to reflect variability in follow-up of individuals, such as loss to follow-up or disenrollment, while making no additional assumptions about the censoring distribution. Together, these settings defined the analytic datasets for each simulation scenario.

Across these simulation settings, we compared model estimates of diagnosis probabilities from 3 categories of approaches: classification model, traditional TTE models, and the mixture cure model.

#### Data analysis

For each analytic dataset generated under the scenarios described above, we first assigned a sequence of administrative censoring times to mimic alternative study cutoffs. This design allowed us to examine how estimates from different models change when the analytic window is truncated at earlier versus later times. We then applied 4 analytic models to each dataset to estimate predicted probabilities: logistic regression, Cox PH, log-normal parametric survival model, and the mixture cure model. For logistic regression, the outcome was defined as ever vs never diagnosed by the censoring time. The Cox PH model and the log-normal parametric survival model were fit to time-to-diagnosis with censoring. For the mixture cure model, we used the R-package *flexsurvcure*. The latency component was specified as a log-normal survival model to represent the distribution of event times among susceptible individuals, and the incidence component was specified as a logistic regression model to estimate the probability of belonging to the susceptible group. We selected the log-normal distribution for the latency component both because it flexibly represents skewed diagnostic timing and because event times in the simulation study were generated from a log-normal distribution. Using the same distribution ensured consistency with the data-generating process and allowed for direct comparison with a log-normal parametric survival model. The group indicator was included as the covariate in all models and in both components for the mixture cure model. For each model, we calculated the predicted probability of diagnosis in each scenario and for every group. Specifically, for the Cox PH and log-normal survival models, the diagnostic probability was defined as $1-S(t_{c})$, where $S(t_{c})$ is the model-predicted survival probability at the administrative censoring time. In the mixture cure model, the proportion classified as the “uncured” directly represented the probability of diagnosis. We note that any difference between the 2 exposure groups indicates bias.

### Real data analysis

#### Data set-up

To illustrate how the mixture cure model provides less biased results regarding probabilities of diagnosis in real data, we used North Carolina Medicaid professional and institutional claims data from 2014 to 2023, with the intent of predicting an autism diagnosis. We restricted the cohort to 59 456 children born in 2014 who were enrolled in Medicaid within 1-month of birth and had continuous enrollment through 18 months. Follow-up data were available through 2023, providing coverage up to age 10. These data provide a few advantages. As an administrative claims-based data source, all diagnoses, regardless of care setting, would be captured. Moreover, as a single payer data source all children should be able to attain similar services. However, as a statewide data source, we expect there to be variability to access to care and opportunity for diagnoses. Finally, by having longer follow-up we have increase our fidelity of capturing an autism diagnosis. We note that the median age of autism diagnosis is 49 months (approximately 4 years old),^14^ suggesting that most children who are autistic, would be expected to have received a diagnosis by age 10. We applied a widely used computable phenotype for autism, requiring 2 diagnoses from a set of ICD-codes.^15^ We used the time of the first code as the time-to for the outcome. We censored children when they lost Medicaid coverage or at the end of the database. To assess potential for bias across race and ethnicity, we used each child’s reported race/ethnicity classification of non-Hispanic White, non-Hispanic Black and Hispanic, which together represented the majority of the dataset. For analytic purposes, we dropped 9421 children who did not fit into one of these groups because their small sample sizes and very few observed cases in each group would have produced unstable estimates in the mixture cure framework. This resulted in a final analytic sample of 50 035 children. As our objective was to compare statistical methods rather than to make population-level inference, this restriction does not bias our methodological conclusions, although it does limit generalizability to smaller racial/ethnic groups.

#### Ethical considerations

This study was approved by the Duke University Institutional Review Board (Pro00111373) and the North Carolina Department of Health and Human Services, with data use agreements in place.

#### Data analysis

We fit a Kaplan-Meier (KM) curve for time-to-diagnosis stratified on race-ethnicity. To illustrate the potential for bias, we set the administrative censoring time at different age horizons and applied the same set of analytic models (logistic regression, Cox PH, log-normal survival model, and mixture cure model) to the dataset, as described in the Simulation Study section. For the mixture cure model, we again used the R package *flexsurvcure*, specifying a log-normal survival model for latency and logistic regression for incidence to ensure consistency with the simulation study. Race/ethnicity (non-Hispanic White, non-Hispanic Black, Hispanic) was included as the covariate in all models and in both components for the mixture cure model. We estimated the predicted diagnostic probability. To assess the variability of these predictions, we bootstrapped each analytic dataset 1000 times, deriving 95% confidence intervals (CIs) for each set of diagnostic probabilities.

All analyses were conducted in R4.1.3.^16^

## Results

### Simulation results

**Figure 1.**
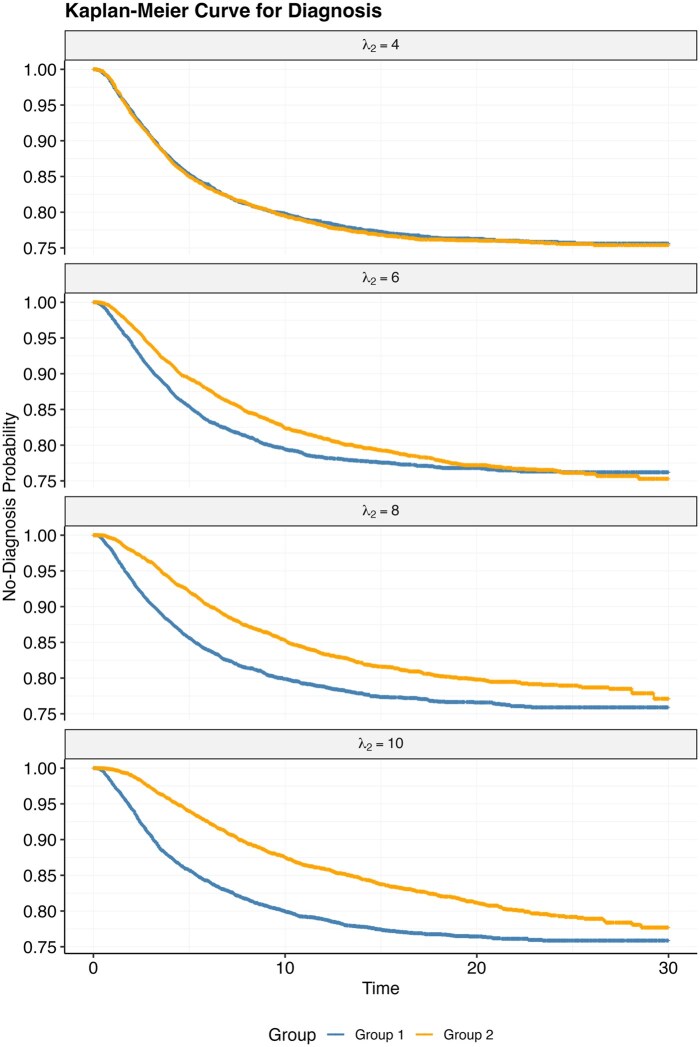
Kaplan-Meier curve for diagnosis of 2 groups under the simulation model.

**Figure 2.**
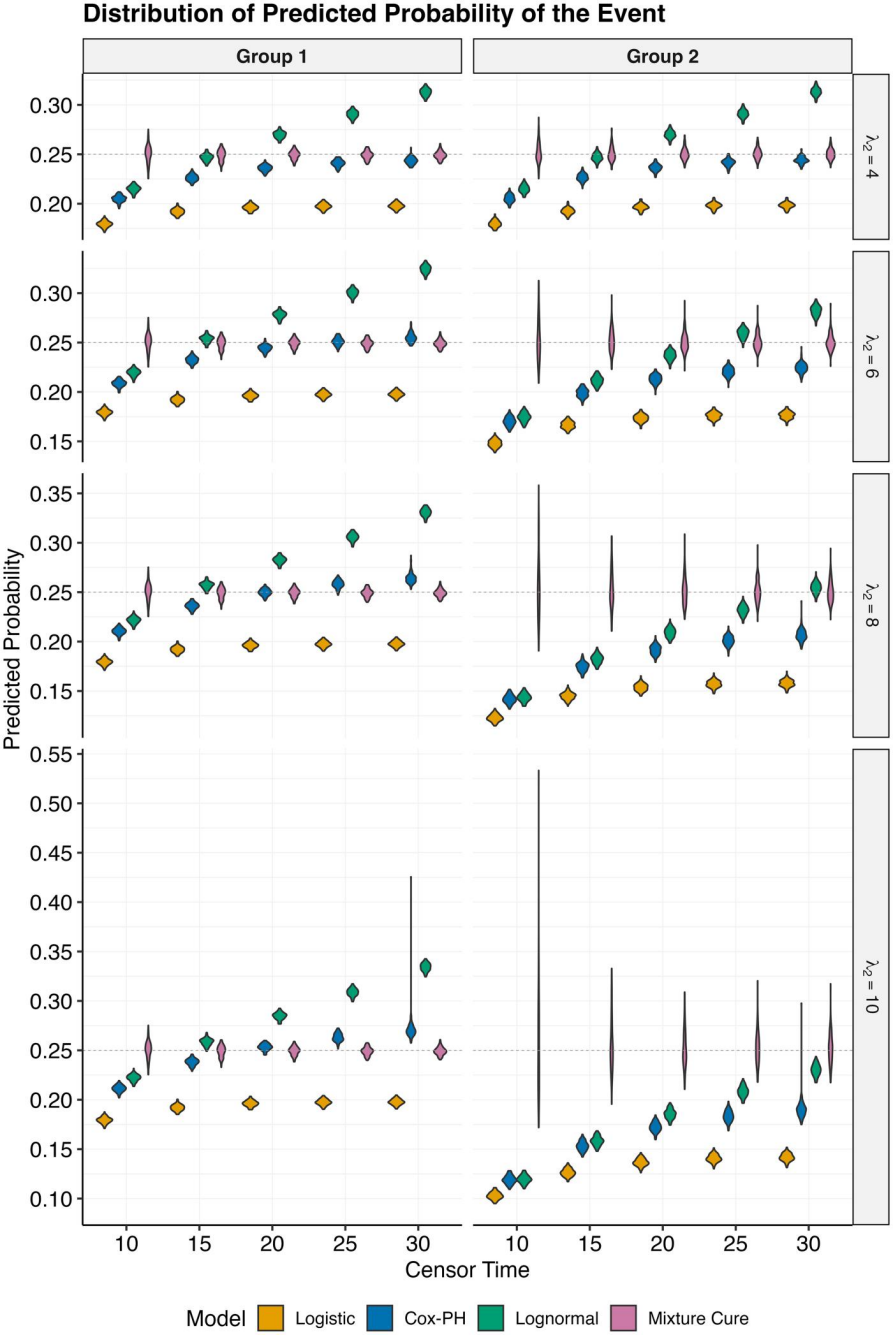
Distribution of predicted probabilities of the event for 2 groups under different modeling strategies. The dashed line indicates the true event rate (25%). With earlier censoring times, the logistic and Cox models have biased estimates while the mixture cure model consistently estimates the correct event rate.

Figure 1 illustrates a KM curve for a simulated dataset for each of the 4 scenarios before the administrative censoring, for the “high” rate event. We note that while the KM curves initially diverge as the difference in median time ($\lambda_{2}$) increases, they all converge to the same overall event rate (25%). Figure 2 shows the estimated predicted probabilities of 2 groups for each dataset and each model. We note that logistic regression under-estimates the event rate, due to the censoring. When the event rates across groups are the same (Figure 2, $\lambda_{2}$ = 4), all models produce equivalent estimates for 2 groups (ie, no differential bias). As the difference in median time value between groups rises, results from logistic regression and TTE models exhibit increased bias—estimated probabilities for group 2 are lower than group 1. This effect is most pronounced when there is earlier administrative censoring. Conversely, the mixture-cure model consistently yields unbiased estimates, indicated by the probability gravitating around the true event rate 0.25. However, it is important to note an uptick in result variance concurrent with rising median times and earlier censoring time. Results were similar, though less pronounced in the “low” event rate scenario (Figure S1).

### Real data results

**Figure 3.**
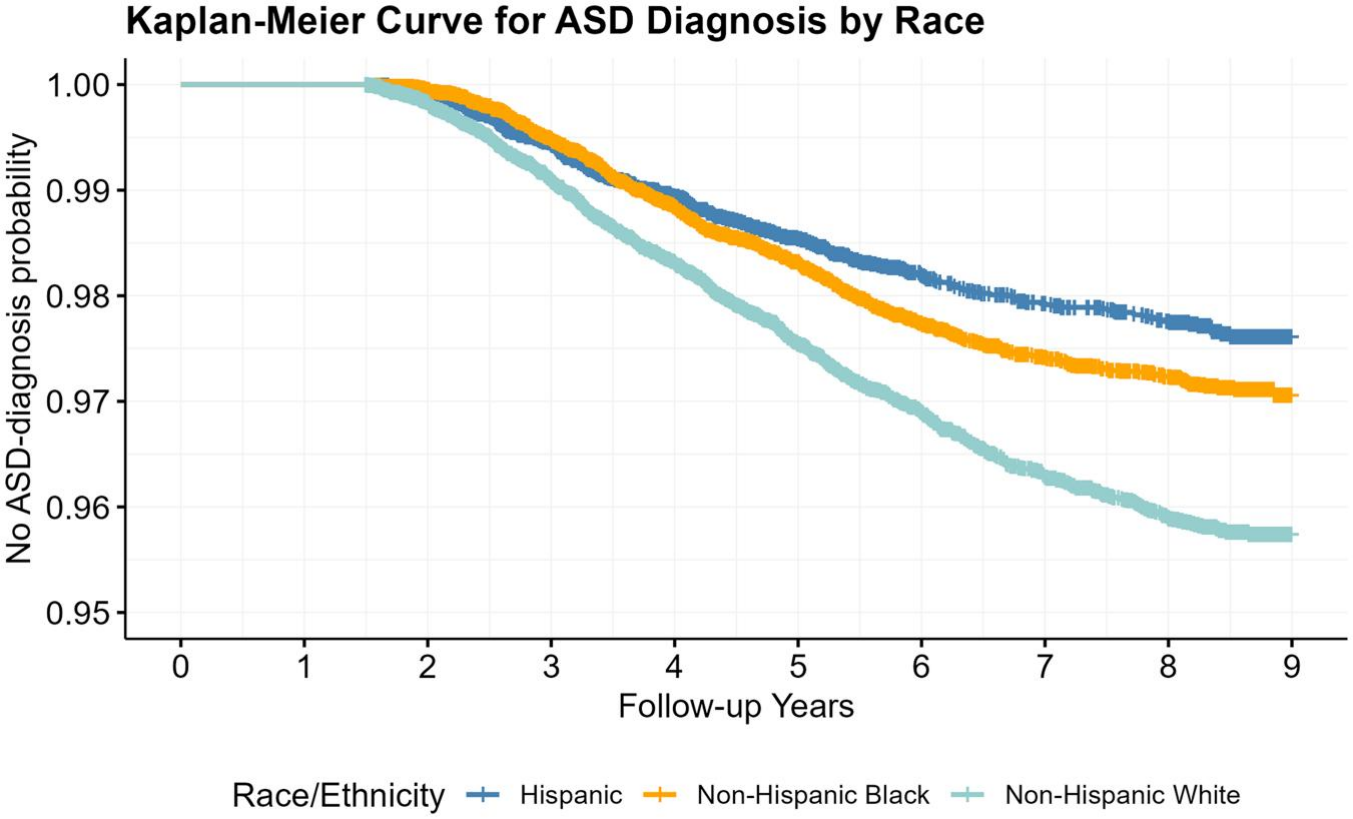
Kaplan-Meier curve for autism diagnosis by race in real data. Diagnosis rates stabilize by 8 years of age. Non-Hispanic Whites have the highest diagnosis rate.

We next present results from the Medicaid claims data. Figure 3 shows the KM curve for time-to-diagnosis across racial-ethnic groups. We note that by age 8 years, all curves have leveled, suggesting that a saturated diagnosis rate is attained. Additionally, we do observe differences in overall diagnosis rates across racial-ethnic groups, with diagnosis more frequent in individuals classified as non-Hispanic White. For analytic purposes, we used the observed diagnosis rate at age 9 as a pragmatic benchmark for each race/ethnicity group, since it was the maximum follow-up age in our dataset.

**Figure 4.**
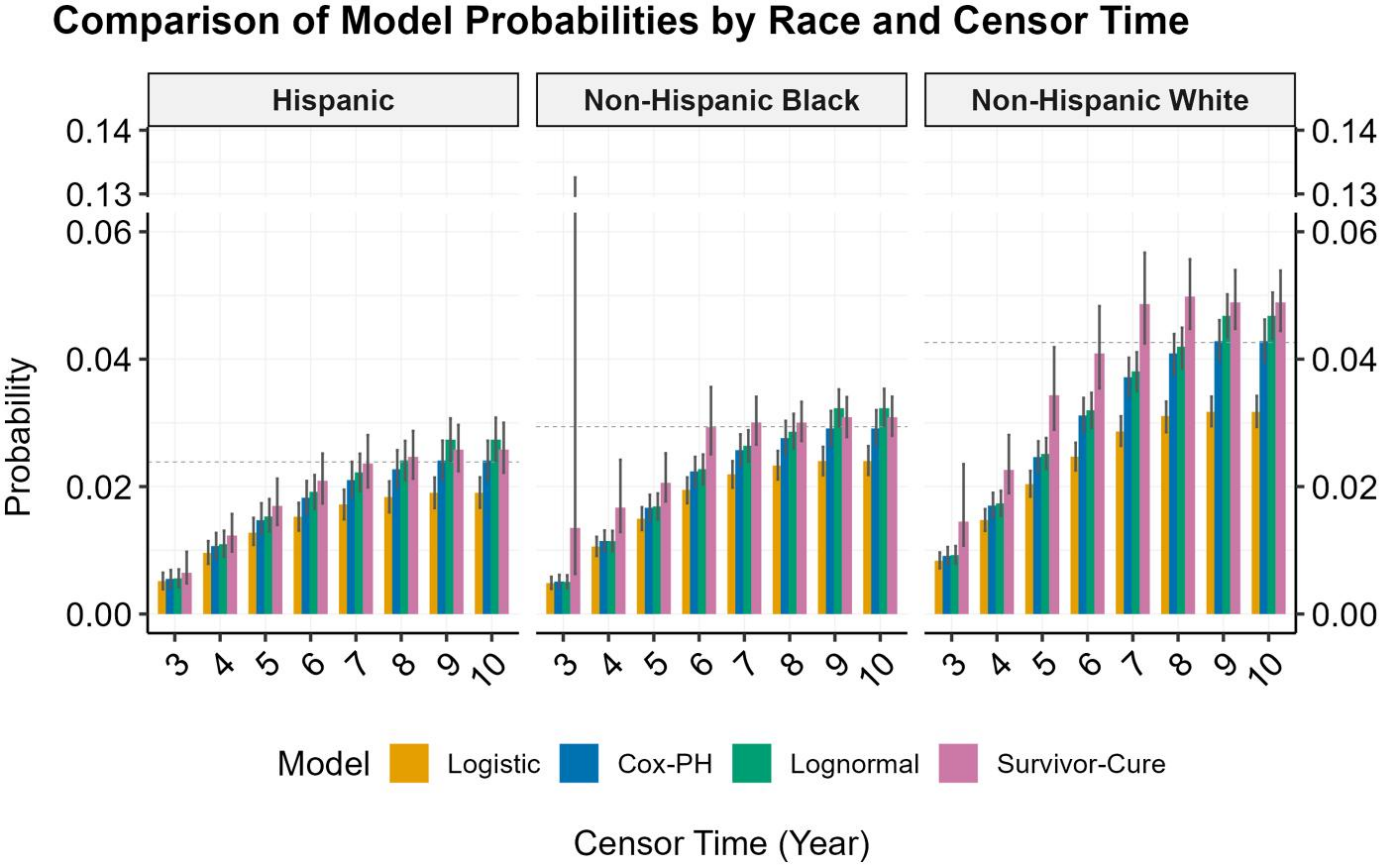
Predicted probability of autism diagnosis in real data. The dashed line indicates the survival probability from the KM curve at the end of the follow-up for the corresponding race and ethnicity. The error bar represents the 95% CI for each probability estimate.

Figure 4 shows the predicted diagnosis probabilities after applying different administrative censoring times to the dataset and applying each of the 4 models. While all models under-estimated the event probability under early censoring, the mixture cure model does produce the largest predicted probabilities closest to the “truth,” and its error bar covers the “truth” earlier than other models. This effect is especially pronounced in the non-Hispanic Black group. While significant variation in initial predictions from the mixture cure model is observed when censoring occurs early (ie, at 3 years), these predictions align closely with the observed diagnosis rate once the censor time extends to 6 years.

## Discussion

Our study is motivated by the goal of predicting autism diagnoses in children. We evaluate the efficacy of the mixture cure model against traditional logistic regression and TTE models for predicting the probability of a binary outcome using TTE data. Our findings highlight the potential for algorithmic bias when using traditional analytic models. Specifically, when there are differences in diagnostic timing between different groups, TTE models yield different probability estimates (ie, bias) across groups while classification models fail to manage censoring. The mixture cure model shows promise in providing unbiased estimates across varying censor times, which is crucial given discrepancies in the time-to-diagnosis due to access to diagnostic services.^8–11^

In traditional applications, mixture cure models focus on inference for the “uncured” group—identifying factors that influence survival, often in cancer studies of long-term outcomes and treatment efficacy.^17–20^ This is done through estimating and drawing inference on the coefficient vectors. In contrast, our interest is prediction, with an emphasis on who will have the event rather than when. We therefore focus on the classification component, centering on accurate probability estimates, rather than the survival component. This shift allows us to address different research questions, moving from what factors influence survival to what proportion of the population has a higher likelihood of a diagnosis.

Overall, our results show the mixture cure model is well suited for this task. The simulation study revealed that logistic regression and TTE models are susceptible to increased bias as diagnostic timing differences grow. Although the mixture cure model produces greater variability (across simulations) and higher variances estimates (analytically) when data are censored early, it produces estimates close to the actual event rate, highlighting its overall robustness. In the application to North Carolina Medicaid data, we observed racial and ethnic disparities in autism diagnostic rates. This variation underscores the necessity for models like the mixture cure model that can adjust for such disparities and provide equitable diagnostic predictions.

Understanding and mitigating algorithmic bias is a primary concern for developers of CPMs. In real-world data such as EHR and claims, algorithmic bias often arises from differential capture of outcomes.^4^ It has been recognized that the informative nature of what we observe in EHR systems can generative informative presence bias^21^^,^^22^ and impact prediction models.^23^ This work highlights another form of informative presence: differential diagnostic timing. Traditional analytic models inflate outcome prevalence among those diagnosed earlier, likely perpetuating access-to-care advantages of certain groups. As other have shown, when CPMs are learned from biased data, they can result in algorithmic unfairness.^2^

In our use case, early autism diagnosis is essential as it connects children with services improving long-term developmental outcomes.^24^ However, differences in access to care influence when children are diagnosed,^11^^,^^25^^,^^26^ creating potential for algorithmic bias in CPMs trained on real-world data. Of note, the most recent study suggests higher diagnosis rates among Hispanic and non-Hispanic Black children compared to non-Hispanic White children.^27^ While our data showed the opposite pattern, this is based on Medicaid claims data thus not nationally representative. Importantly, the mixture cure framework is applicable to outcomes where the focus is less on the timing of the event and instead on whether the event occurs.

The parametric nature of the survivor component is both a feature and a limitation of the mixture cure model. While it offers a structured approach to estimate survival times, this parametric framework can impose constraints that may not align with the complex and heterogeneous nature of certain datasets. The model’s efficacy is contingent upon the fit of the chosen distribution to the actual data; if the distribution does not adequately capture the underlying survival dynamics, the model may yield less accurate or even misleading predictions. The lack of flexibility could limit the model’s application in epidemiological studies that feature complex survival patterns or require nonparametric methods to more accurately capture the nuances of the data. The parametric model, by its nature, also limits the complexity of the parameter vector to generate accurate estimates of the outcome. However, just as logistic and Cox PH models have widely used deep learning analogues (ie, multilayer perceptron classifier and DeepSurv,^28^ respectively), the discrete failure time model can be seen as the deep learning analogue of the mixture cure model due to its ability to estimate both when and whether an event will occur without placing strong assumptions on the form of the survival distribution. In some cases, it may be desirable to explicitly disentangle factors affecting these 2 predictions,^29^ which may help address challenges of differential diagnostic timing and algorithmic bias. We leave this direction for future work.

While we have highlighted the potential of the mixture cure model, this study has some limitations. The simulation study we conducted was intentionally simplified, using only one grouping variable of interest and a simple parametric survival function, in order to clearly illustrate the methodological issue of bias from diagnostic timing. We acknowledge that this does not reflect the complexity of real-world CPMs. Future work should consider the performance of these models in more complex and realistic scenarios. Moreover, the results showed that the most pronounced effects were in the high event rate setting, which is a higher event rate than typically observed in autism diagnoses. In the low event-rate scenario, we observed a higher degree of variability in the estimated probabilities compared to the group differences (Figure S1). While our study focused on autism as a motivating example, future work should extend this framework to other disease contexts, which represents an important next step in evaluating its robustness and generalizability. Researchers and practitioners should be cognizant of these limitations when choosing to employ the mixture cure model and should consider supplementing it with nonparametric techniques or exploring alternative models where appropriate to enhance the robustness of their predictions.

## Conclusion

Our study supports our assertion that the mixture cure model could serve as a valuable tool in predictive modeling, especially when the outcome of interest is not observed uniformly across groups. While there are limitations, its application could enhance the accuracy of clinical predictions and aid in the timely and equitable identification of children diagnosed with autism. Further research should investigate the application of the mixture cure model across other datasets and conditions to validate its efficacy. Additionally, future work could explore the development of more flexible parametric forms for the survivor component, enhancing the model’s utility across various clinical scenarios.

## Supplementary Material

ooaf148_Supplementary_Data

## Data Availability

The data used in this study come from North Carolina Medicaid records, which contain sensitive and identifiable health information. These data are not publicly available. All code to reproduce the simulation study is available at GitHub (https://github.com/p3nny-wu/Mixture_Cure) and archived on Zenodo (DOI: 10.5281/zenodo.17355296).
