# Supplementary material for: Using mixture cure models to address algorithmic bias in diagnostic timing: autism as a test case
Source: JAMIA Open. 2025 Nov 4;8(6):ooaf148. doi: 10.1093/jamiaopen/ooaf148 (PMC12598640; doi:10.1093/jamiaopen/ooaf148)
Supplement: ooaf148_Supplementary_Data [file ooaf148_supplementary_data.docx]

**SUPPLEMENTAL MATERIEL**


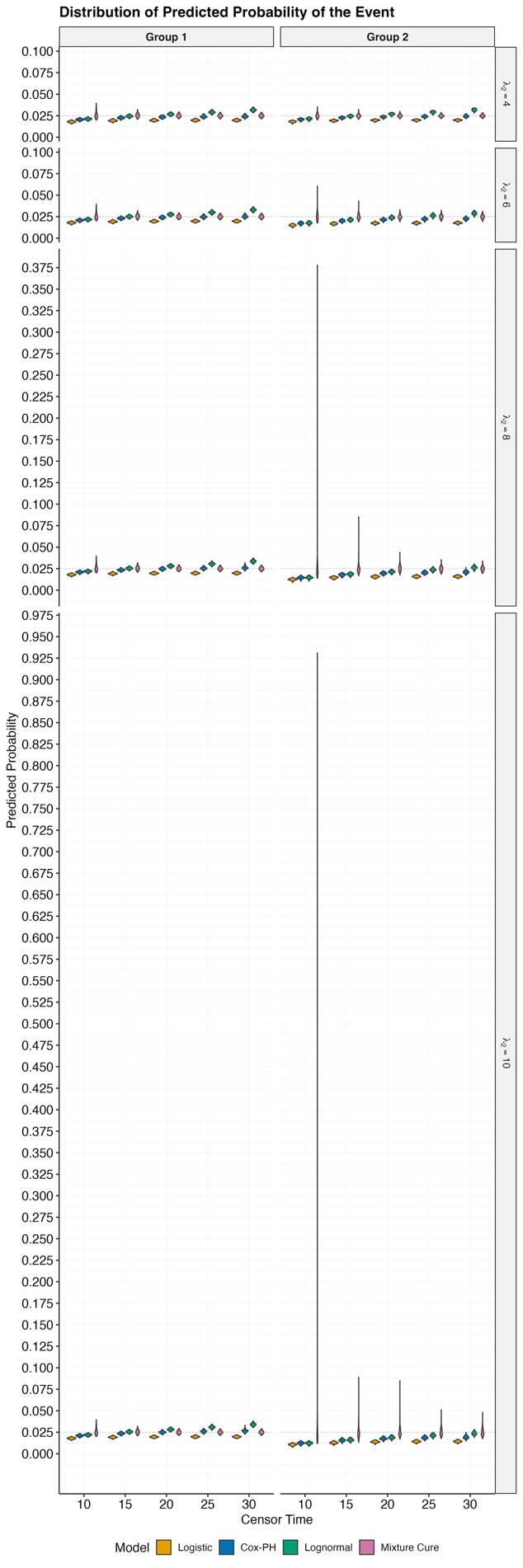


Supplemental Figure 1. Distribution of predicted probabilities of the event for two groups. The dashed line indicates the true event rate (2.5%).
